# Association between Endothelial Activation and Stress Index (EASIX) and uric acid levels: A cross-sectional analysis based on the NHANES

**DOI:** 10.1097/MD.0000000000050443

**Published:** 2026-09-04

**Authors:** Huanyu Wu, Hong Tang, Meng Liu, Yujuan Zhu, Yonghui Li

**Affiliations:** a Department of Orthopedics, Affiliated Hospital (Clinical College) of Xiangnan University, Chenzhou, Hunan Province, PR China.

**Keywords:** cross-sectional study, Endothelial Activation and Stress Index (EASIX), hyperuricemia, NHANES, uric acid

## Abstract

Elevated serum uric acid is strongly associated with metabolic abnormalities and an increased risk of cardiovascular diseases. Endothelial dysfunction is considered a critical link in metabolic abnormalities. The Endothelial Activation and Stress Index (EASIX), a composite laboratory-based index, has not been systematically examined for its association with serum uric acid levels and endothelial function. This study utilized data from the 2007–2018 National Health and Nutrition Examination Survey (NHANES) with a cross-sectional design to assess the relationship between the EASIX index and serum uric acid levels. Multivariable linear regression analysis was conducted to evaluate their independent association, while restricted cubic splines (RCS) and piecewise regression models were employed to explore nonlinear dose–response trends. Subgroup and sensitivity analyses were performed to validate the robustness of the results. This study included 7602 participants, comprising those with normal and elevated serum uric acid levels. Regression analysis revealed a significant positive correlation between the EASIX index and serum uric acid levels after full adjustment for covariates (β = 0.26, 95% CI: 0.06–0.46). Quartile trend analysis demonstrated that serum uric acid levels increased with rising EASIX. RCS analysis revealed a nonlinear relationship, with serum uric acid levels plateauing at an EASIX value of approximately 1.31, suggesting a “threshold saturation” effect. Subgroup analyses confirmed consistent results across different demographic characteristics and disease statuses, and sensitivity analyses further validated the robustness of the findings. Among US adults, EASIX was positively and nonlinearly associated with serum uric acid levels. Exploratory analysis further suggested that higher EASIX quartiles were associated with increased odds of hyperuricemia. These findings indicate that EASIX may reflect systemic endothelial stress and metabolic burden related to uric acid variation. Further prospective and externally validated studies are needed to determine its clinical utility.

## 1. Introduction

Elevated serum uric acid and hyperuricemia are common metabolic abnormalities resulting from increased purine metabolism and decreased uric acid excretion, characterized by sustained or recurrent elevations in serum uric acid levels.^[1]^ Uric acid, the end product of human purine metabolism, possesses antioxidant properties at physiological concentrations.^[2]^ However, when elevated abnormally, it exerts adverse effects on multiple organ systems through mechanisms such as inducing oxidative stress and activating inflammatory signaling pathways.^[3]^ In recent years, with the aging population and the rising prevalence of obesity, the burden of hyperuricemia has significantly increased.^[4,5]^ Numerous epidemiological studies have shown that hyperuricemia is not only the direct pathological basis for gout but also significantly associated with the risk of hypertension, diabetes, and chronic kidney disease.^[6]^ Notably, even in populations where the diagnostic threshold for hyperuricemia has not been reached, a progressive increase in serum uric acid levels within the normal high range has been closely associated with impaired cardiovascular and renal function, as well as metabolic disorders.^[7]^ Given the increasing long-term medical burden and socio-economic costs of hyperuricemia-related diseases, systematically identifying quantifiable biological characteristics underlying elevated uric acid levels is of significant public health importance for early risk stratification and comprehensive prevention. However, the current understanding of the relationship between elevated uric acid levels and subclinical systemic damage remains insufficient, particularly regarding its association with endothelial dysfunction. A large-scale, population-based systematic assessment is still lacking.

Endothelial dysfunction is widely recognized as a key pathological link between metabolic abnormalities and adverse cardiovascular and renal outcomes.^[8]^ Elevated uric acid levels directly damage endothelial cell function through mechanisms such as inhibiting endothelial nitric oxide synthase (eNOS), enhancing reactive oxygen species (ROS) generation, and promoting inflammation, leading to microcirculatory disturbances, organ perfusion abnormalities, and disruption of vascular homeostasis.^[9,10]^ Therefore, elevated uric acid impacts vascular health beyond metabolic abnormalities and affects the homeostasis of multiple systems throughout the body. In this context, a comprehensive and systematic quantifiable index reflecting endothelial activation and stress holds significant research value. Endothelial Activation and Stress Index (EASIX) is a composite index composed of lactate dehydrogenase (LDH), serum creatinine (SCR), and platelet count (PLT), designed to reflect endothelial stress from multiple dimensions. EASIX is a composite laboratory-based index calculated from lactate dehydrogenase, serum creatinine, and platelet count. It was originally developed in clinical settings characterized by endothelial injury and thrombotic microangiopathy. EASIX has therefore been proposed as an indirect and nonspecific surrogate of systemic endothelial and microvascular stress.^[11,12]^ Existing studies have demonstrated the significant value of EASIX in risk assessment and prognosis prediction across various clinical diseases. For example, EASIX has been widely applied in evaluating the prognosis of hematopoietic stem cell transplantation-related complications, graft-versus-host disease, severe infections, and cancer patients, and is closely related to cardiovascular events and mortality risk.^[13-16]^ However, despite some research on EASIX’s application in these diseases, systematic epidemiological evidence regarding its relationship with elevated uric acid levels is still lacking. Given the key role of elevated uric acid in endothelial damage, this study aims to systematically evaluate the relationship between EASIX and serum uric acid levels using the National Health and Nutrition Examination Survey (NHANES) database, enhancing the biological understanding of uric acid-related endothelial stress and exploring the epidemiological association between EASIX and uric acid-related metabolic changes. This study aims to provide new theoretical foundations for early screening, risk assessment, and individualized intervention strategies for uric acid-related diseases.

## 2. Materials and methods

### 2.1. Data source and study design

This study uses the NHANES database and is a nationally representative cross-sectional study. NHANES is organized and conducted by the National Center for Health Statistics (NCHS) using a complex, stratified, multi-stage probability sampling design to systematically investigate demographic characteristics, lifestyle, physical examinations, and laboratory test indicators of the non-institutionalized US population. All survey procedures were approved by the NCHS Institutional Review Board, and written informed consent was obtained from all participants before the survey.

**Figure 1.**
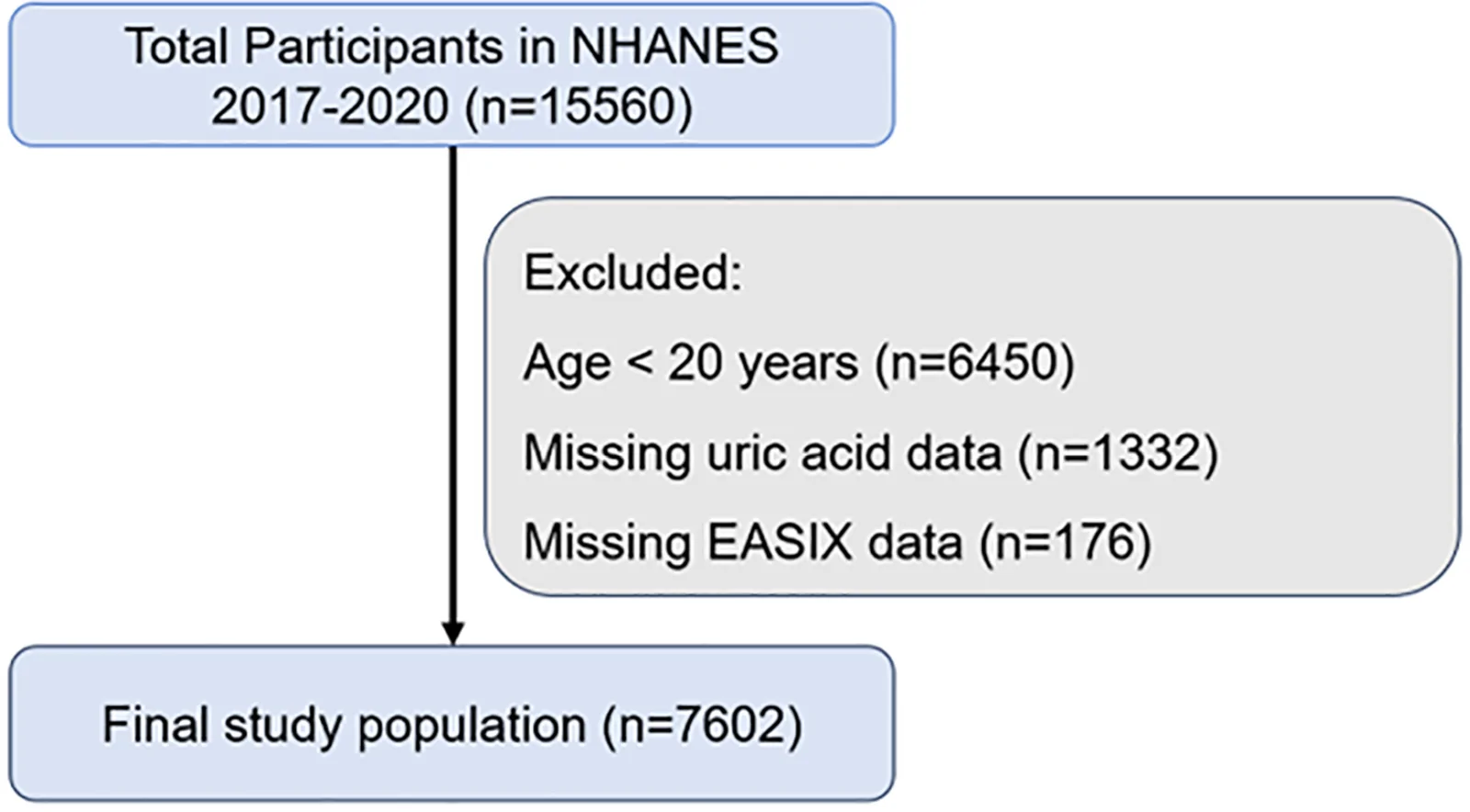
Participant selection and inclusion process. EASIX = Endothelial Activation and Stress Index, NHANES = National Health and Nutrition Examination Survey.

Participants were excluded if they were younger than 20 years or had missing data on serum uric acid, lactate dehydrogenase, serum creatinine, platelet count, or key covariates (Fig. 1). Participants were not excluded based on recent dietary intake because NHANES is an observational survey and does not provide controlled pre-examination dietary restriction data for all participants.

### 2.2. Definition of EASIX

EASIX is a composite laboratory-based index that has been proposed as an indirect surrogate of systemic endothelial and microvascular stress. It is calculated using serum LDH, serum creatinine, and platelet count as follows:


$$EASIX=\frac{LDH\;\;\;\times \;\;\;Scr}{PLT}.$$

LDH (U/L), Scr (mg/dL), and PLT (×10^9^/L) are derived from NHANES standardized laboratory data. EASIX was included as a continuous variable in regression models and categorized into quartiles (Q1–Q4) for descriptive analysis and group comparisons.

### 2.3. Measurement of serum uric acid

Serum uric acid levels were measured using the Beckman UniCel® DxC800 Synchron or Beckman Synchron LX20 automated biochemical analyzers (Beckman Coulter Inc.). The detection principle is based on the oxidation of uric acid to produce allantoin and hydrogen peroxide. Serum uric acid levels were the primary outcome variable in the study, included as a continuous variable in regression models, and categorized by weighted quartiles to evaluate the dose–response relationship with EASIX. In exploratory analyses, hyperuricemia was additionally defined as serum uric acid >7.0 mg/dL in men and >6.0 mg/dL in women. Logistic regression models were used to evaluate the association between EASIX and hyperuricemia.

### 2.4. Definition of covariates

To minimize potential confounding factors, a series of covariates related to both serum uric acid levels and endothelial function were included in the multivariable models. Demographic variables included age, sex, and race; socioeconomic factors included the poverty-income ratio (PIR) and education level; lifestyle factors included smoking status and physical activity level; and clinically relevant variables included body mass index (BMI), diabetes, hypertension, and chronic kidney disease (CKD). Smoking status was classified based on NHANES questionnaire data; physical activity level was assessed based on self-reported moderate and vigorous physical activity; and diabetes, hypertension, and CKD were defined based on self-reported physician diagnoses, relevant medication use, or standardized questionnaire data.

### 2.5. Statistical analysis

Descriptive statistics were used to analyze the baseline characteristics of the study participants. Continuous variables were expressed as weighted means ± standard deviation, and categorical variables as weighted frequencies and percentages, with group comparisons based on EASIX quartiles. Multivariable linear regression models were used to evaluate the association between EASIX and serum uric acid levels, with the following stepwise adjustment models: unadjusted model (model 1), model adjusted for age, sex, and race (model 2), and a fully adjusted model (model 3) further adjusted for BMI, PIR, education level, smoking status, physical activity level, diabetes, hypertension, and CKD. Restricted cubic spline (RCS) analysis was used to explore potential nonlinear relationships between EASIX and serum uric acid levels. A 2-stage linear regression model was used to assess threshold effects, and goodness-of-fit between the linear and piecewise models was compared using likelihood ratio tests. Stratified analyses were performed in subgroups to assess the consistency of the results, and multiple sensitivity analyses were conducted to validate the robustness of the findings. All statistical analyses considered NHANES’ complex sampling design and applied the appropriate sampling weights. Analyses were conducted using R software (version 4.2.3), with a 2-sided *P*-value < 0.05 considered statistically significant.

## 3. Results

### 3.1. Baseline characteristics

A total of 7602 participants were included, and their baseline characteristics are presented in Table 1, categorized by EASIX quartiles. As EASIX levels increased, the proportion of participants aged > 50 years and males significantly increased. Higher EASIX groups showed a significantly higher prevalence of obesity, hypertension, diabetes, and chronic kidney disease, suggesting that populations with higher systemic endothelial stress levels exhibit more unfavorable metabolic and cardiovascular risks. Laboratory indicators showed that serum uric acid levels increased with higher EASIX quartiles. These results suggest a significant positive correlation between EASIX and serum uric acid.

**Table 1:** Baseline characteristics of the study population.

Characteristic	Group	Overall	EASIX				*P*-value
			Q1 (<0.40)	Q2 (0.40–0.55)	Q3 (0.55–0.76)	Q4 (>0.76)	
N		7602	1901	1901	1899	1901	
Age (%)	>50	4030 (53.0)	639 (33.6)	934 (49.1)	1062 (55.9)	1395 (73.4)	<.001
	20–50	3572 (47.0)	1262 (66.4)	967 (50.9)	837 (44.1)	506 (26.6)	
Sex (%)	Female	3956 (52.0)	1605 (84.4)	1083 (57.0)	732 (38.5)	536 (28.2)	<.001
	Male	3646 (48.0)	296 (15.6)	818 (43.0)	1167 (61.5)	1365 (71.8)	
Race (%)	Mexican American	901 (11.9)	327 (17.2)	253 (13.3)	187 (9.8)	134 (7.0)	<.001
	Non-Hispanic Black	1924 (25.3)	389 (20.5)	415 (21.8)	491 (25.9)	629 (33.1)	
	Non-Hispanic White	2698 (35.5)	548 (28.8)	692 (36.4)	707 (37.2)	751 (39.5)	
	Others	2079 (27.3)	637 (33.5)	541 (28.5)	514 (27.1)	387 (20.4)	
BMI (%)	Normal	1751 (23.4)	434 (23.1)	467 (24.9)	450 (24.1)	400 (21.5)	.003
	Obese	5640 (75.4)	1408 (75.1)	1386 (73.9)	1396 (74.9)	1450 (77.9)	
	Underweight	87 (1.2)	34 (1.8)	23 (1.2)	19 (1.0)	11 (0.6)	
Education level (%)	Above high school	4357 (57.3)	1102 (58.0)	1099 (57.8)	1112 (58.6)	1044 (54.9)	.001
	High school or equivalent	1806 (23.8)	394 (20.7)	450 (23.7)	452 (23.8)	510 (26.8)	
	No record	12 (0.2)	3 (0.2)	2 (0.1)	2 (0.1)	5 (0.3)	
	Under high school	1427 (18.8)	402 (21.1)	350 (18.4)	333 (17.5)	342 (18.0)	
PIR, mean (SD)		2.62 (1.62)	2.51 (1.63)	2.67 (1.64)	2.65 (1.63)	2.64 (1.60)	.032
Activity status (%)	Active	4253 (55.9)	1002 (52.7)	1084 (57.0)	1116 (58.8)	1051 (55.3)	.001
	Inactive	3349 (44.1)	899 (47.3)	817 (43.0)	783 (41.2)	850 (44.7)	
Smoke (%)	No	4394 (57.8)	1218 (64.1)	1142 (60.1)	1038 (54.7)	996 (52.4)	<.001
	Yes	3204 (42.1)	682 (35.9)	757 (39.8)	860 (45.3)	905 (47.6)	
	No record	4 (0.1)	1 (0.1)	2 (0.1)	1 (0.1)	0 (0.0)	
Diabetes (%)	No	6166 (81.1)	1589 (83.6)	1583 (83.3)	1590 (83.7)	1404 (73.9)	<.001
	Yes	1198 (15.8)	261 (13.7)	257 (13.5)	250 (13.2)	430 (22.6)	
	No record	238 (3.1)	51 (2.7)	61 (3.2)	59 (3.1)	67 (3.5)	
Hypertension (%)	No	4619 (60.8)	1341 (70.5)	1197 (63.0)	1179 (62.1)	902 (47.4)	<.001
	Yes	2971 (39.1)	554 (29.1)	703 (37.0)	718 (37.8)	996 (52.4)	
	No record	12 (0.2)	6 (0.3)	1 (0.1)	2 (0.1)	3 (0.2)	
CKD (%)	No	6850 (90.1)	1929 (91.6)	1685 (90.8)	1605 (89.3)	1631 (88.5)	.024
	Yes	733 (9.6)	172 (8.2)	166 (8.9)	186 (10.4)	209 (11.3)	
	No record	19 (0.2)	5 (0.2)	5 (0.3)	6 (0.3)	3 (0.2)	
Hyperuricemia (%)	No	6194 (81.5)	1708 (89.8)	1631 (85.8)	1525 (80.3)	1330 (70.0)	<.001
	Yes	1408 (18.5)	193 (10.2)	270 (14.2)	374 (19.7)	571 (30.0)	
PLT, mean (SD) (×10^9^/L)		245.64 (65.70)	306.18 (65.07)	256.12 (47.32)	228.19 (41.31)	192.05 (46.20)	<0.001
LDH, mean (SD) (U/L)		159.04 (34.95)	140.81 (24.02)	151.97 (24.77)	160.44 (26.33)	182.94 (45.25)	<.001
SCR, mean (SD) (mg/dL)		0.91 (0.53)	0.67 (0.13)	0.81 (0.15)	0.92 (0.17)	1.24 (0.94)	<.001
UA, mean (SD) (mg/dL)		5.41 (1.48)	4.70 (1.26)	5.20 (1.35)	5.61 (1.38)	6.11 (1.52)	<.001

Mean (SD) for continuous variables, % for categorical variables.

BMI = body mass index, CKD = chronic kidney disease, EASIX = endothelial activation and stress index, LDH = lactate dehydrogenase, PIR = Poverty index ratio, PLT = platelet count, SCR = serum creatinine, SD = standard deviation, UA = uric acid.

### 3.2. Association between EASIX and serum uric acid levels

Multivariable linear regression analysis was used to evaluate the relationship between EASIX and serum uric acid (Table 2). In the unadjusted model (model 1), EASIX was significantly positively correlated with serum uric acid (β = 0.55, 95% CI: 0.35–0.76). After adjusting for age, sex, and race (model 2), this association weakened but remained significant (β = 0.26, 95% CI: 0.12–0.40). After full adjustment for covariates (model 3), the positive correlation between EASIX and serum uric acid remained (β = 0.26, 95% CI: 0.06–0.46). When EASIX was categorized into quartiles, serum uric acid levels in higher EASIX groups were significantly elevated compared with the lowest quartile (Q1), showing a clear dose-dependent trend, indicating a stable positive dose–response relationship.

**Table 2:** The relationship between EASIX and uric acid.

		Model 1 β (95% CI) *P*-value	Model 2 β (95% CI) *P*-value	Model 3 β (95% CI) *P*-value
Uric acid	EASIX	0.55 (0.35, 0.76) <.001	0.26 (0.12, 0.40) <.001	0.26 (0.06, 0.46) .024
	Q1	[Reference]	[Reference]	[Reference]
	Q2	0.51 (0.34, 0.67) <.001	0.21 (0.07, 0.35) .006	0.25 (−0.09, 0.58) .086
	Q3	0.94 (0.78, 1.1) <.001	0.44 (0.29, 0.59) <.001	0.46 (0.18, 0.74) .019
	Q4	1.30 (1.10, 1.50) <.001	0.70 (0.48, 0.93) <.001	0.72 (0.23, 1.20) .024
	*P* for trend	<.001	<.001	<.001

Model 1: no covariates adjusted; model 2: adjusted for age, sex, and race; model 3: adjusted for age, sex, race, BMI, PIR, educational level, smoking, activity status, diabetes, hypertension, CKD.

BMI = body mass index, CI = confidence interval, CKD = chronic kidney disease, EASIX = Endothelial Activation and Stress Index, PIR = poverty–income ratio, Q = quartiles.

### 3.3. Nonlinear relationship and threshold effect analysis

**Figure 2.**
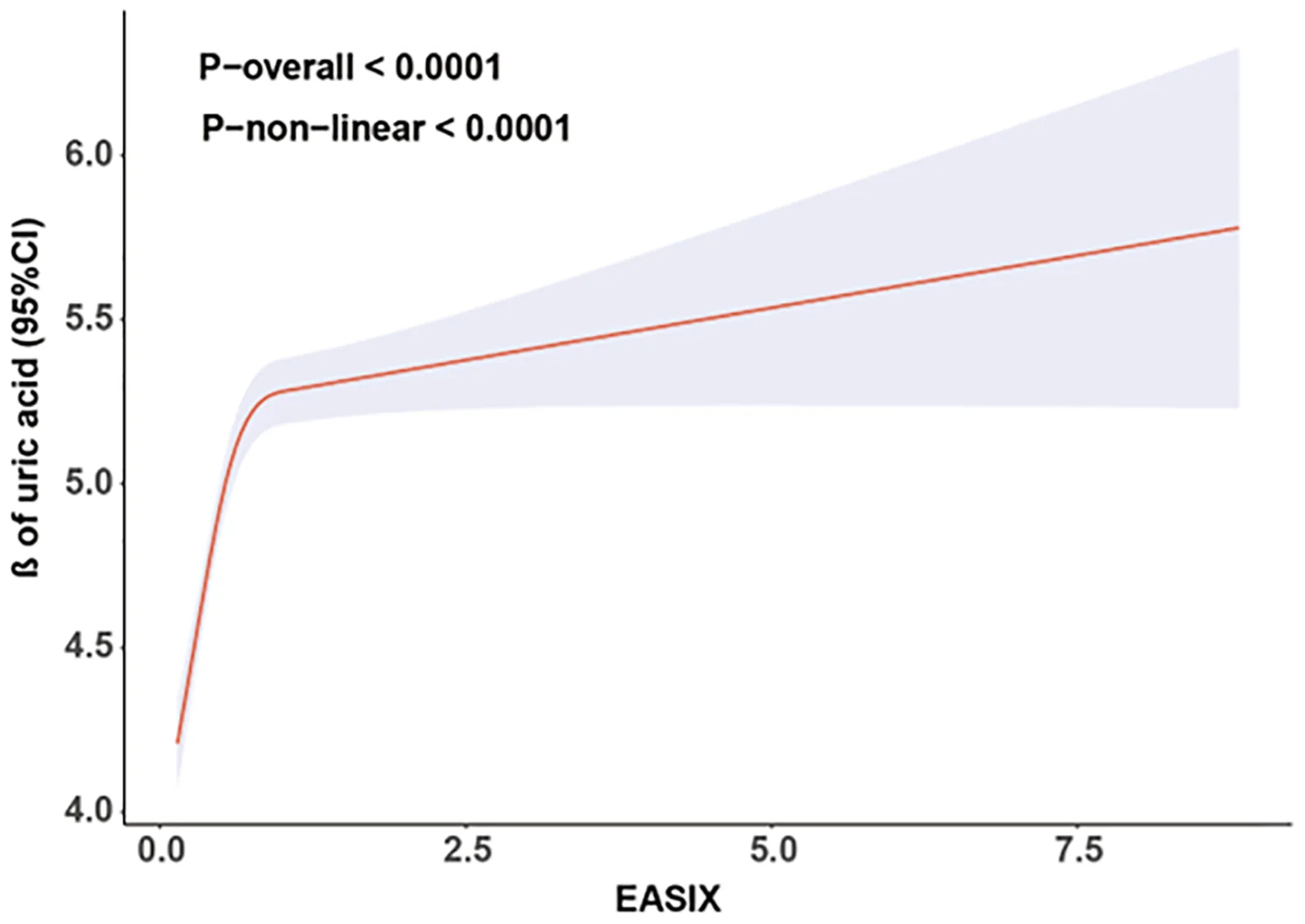
RCS curve fits the association of EASIX with uric acid. Adjusted for age, sex, race, BMI, PIR, educational level, smoke, activity status, diabetes, hypertension, CKD. BMI = body mass index, CI = confidence interval, CKD = chronic kidney disease, EASIX = Endothelial Activation and Stress Index, PIR = poverty–income ratio, RCS = restricted cubic splines.

RCS analysis showed a significant nonlinear relationship between EASIX and serum uric acid (Fig. 2). The overall trend indicated that, within lower to moderate EASIX levels, serum uric acid increased as EASIX rose. Further 2-stage linear regression analysis identified a potential threshold. When EASIX < 1.31, EASIX was significantly positively correlated with serum uric acid (β = 1.10, 95% CI: 1.00–1.30); however, when EASIX ≥ 1.31, the association reversed, becoming negative (β = −0.09, 95% CI: −0.15 to −0.03). Likelihood ratio tests showed that, compared with the single linear model, the piecewise regression model with a threshold fit significantly improved (Table 3), suggesting a potential phase-specific relationship between EASIX and serum uric acid.

**Table 3:** Two-stage linear regression between EASIX and uric acid.

	EASIX	β (95% CI) *P*-value
Uric acid	Standard linear model	0.19 (0.14, 0.24) <.001
	EASIX < 1.31	1.10 (1.00, 1.30) <.001
	EASIX > 1.31	−0.09 (−0.15, −0.03) .004
	Log-likelihood ratio test	<.001

Adjusted for age, sex, race, BMI, PIR, educational level, smoke, activity status, diabetes, hypertension, CKD.

BMI = body mass index, CI = confidence interval, CKD = chronic kidney disease, EASIX = Endothelial Activation and Stress Index, PIR = poverty–income ratio.

### 3.4. Subgroup analysis

To explore potential moderating effects of EASIX on the association between serum uric acid and different populations, stratified subgroup analyses were performed based on the fully adjusted model (model 3), with results presented in Table 4. The analysis showed that the positive correlation between EASIX and serum uric acid was more significant in populations aged > 50 years, males, smokers, those with insufficient physical activity, and individuals with comorbidities of hypertension or diabetes. Interaction analysis further revealed significant interactions between sex and hypertension status in the association between EASIX and serum uric acid, suggesting that this relationship may be heterogeneous across different population characteristics.

**Table 4:** Subgroup analysis between EASIX and uric acid.

Characteristic	Group	OR (95% CI) *P*-value	*P* for interaction
Age	20–50	0.17 (−0.05, 0.38) .110	.800
	>50	0.29 (0.07, 0.52) .019	
Sex	Male	0.14 (0.04, 0.24) .014	<.001
	Female	0.77 (0.34, 1.20) .004	
Smoke	No	0.22 (-0.04, 0.48) .083	.700
	Yes	0.31 (0.07, 0.56) .019	
Activity status	Active	0.23 (−0.03, 0.48) .069	.700
	Inactive	0.30 (0.06, 0.54) .024	
Hypertension	No	0.69 (0.35, 1.00) .003	<.001
	Yes	0.16 (0.02, 0.29) .029	
CKD	No	0.27 (0.06, 0.47) .019	.400
	Yes	0.22 (-0.11, 0.55) .200	
Diabetes	No	0.28 (0.02, 0.55) .039	.120
	Yes	0.14 (0.00, 0.28) .046	

CI = confidence interval, CKD = chronic kidney disease, EASIX = Endothelial Activation and Stress Index, OR = odds ratio.

### 3.5. Sensitivity analysis

In the sensitivity analysis, extreme values of EASIX (±3 SD) were excluded, and the model was rerun, including 7538 participants. The results are shown in Table S1, Supplemental Digital Content 1. The positive association between EASIX and serum uric acid remained consistent after excluding extreme values, supporting the robustness of the main findings. In addition, an exploratory logistic regression analysis using hyperuricemia as a binary outcome showed that higher EASIX levels were associated with increased odds of hyperuricemia, particularly among participants in the higher EASIX quartiles, with a significant trend across quartiles (Table S2, Supplemental Digital Content 2).

## 4. Discussion

This study, based on a nationally representative NHANES sample, explored the association between the EASIX and serum uric acid. The results show a significant positive correlation between EASIX and serum uric acid, which remains significant after multivariable adjustment, indicating an association between EASIX and serum uric acid levels. The association was nonlinear, suggesting that EASIX, a composite laboratory-based index, may provide complementary information related to serum uric acid variation. These results provide new epidemiological evidence suggesting that EASIX may provide complementary information regarding endothelial stress and metabolic burden.

This study used the EASIX as a composite laboratory-based index to evaluate the relationship between EASIX and serum uric acid. Unlike previous studies focusing on the relationship between uric acid and a single metabolic abnormality, this study integrates lactate dehydrogenase, serum creatinine, and platelet count to reflect endothelial stress and metabolic load, filling a gap in existing research.^[17-19]^ A significant nonlinear association was observed between EASIX and serum uric acid. Although severe endothelial injury may reduce circulating platelet counts through platelet consumption, the inverse platelet pattern is also partly inherent to the EASIX formula and does not independently demonstrate endothelial dysfunction. LDH is a nonspecific marker of cellular injury, hypoxia, or hemolysis and should be interpreted as an indirect component of systemic cellular and microvascular stress. At lower to moderate EASIX levels, increased serum uric acid directly damages endothelial cells through mechanisms such as oxidative stress and enhanced inflammatory response, exacerbating endothelial dysfunction.^[20,21]^ The mechanisms underlying this nonlinear association remain uncertain. The change in slope above the estimated threshold may reflect differences in the relative contributions of the EASIX components, residual confounding, reverse causality, or limited observations at the upper end of the EASIX distribution. EASIX, as a multi-biomarker integrated scoring tool, reflects systemic endothelial stress and offers a new perspective for assessing metabolic load. Subgroup analysis further confirmed the stability of the relationship between EASIX and serum uric acid, particularly in high-risk groups such as older individuals, males, smokers, those with insufficient physical activity, and those with comorbid hypertension or diabetes, where the positive correlation between EASIX and serum uric acid was more significant. This suggests that these populations may be more susceptible to endothelial stress, strengthening the potential application value of EASIX in these groups. These results suggest that EASIX may provide complementary information for characterizing systemic endothelial stress and metabolic burden related to serum uric acid variation, supporting further investigation in prospective and externally validated studies.

An increase in the EASIX index reflects the overall metabolic load resulting from impaired endothelial function, renal dysfunction, and cellular metabolic stress, all key triggers for elevated serum uric acid. Pathophysiologically, abnormalities in multiple biological mechanisms can jointly affect endothelial function and renal metabolism, promoting the elevation of serum uric acid. High EASIX levels are typically accompanied by cellular damage, metabolic stress, and endothelial injury, promoting uric acid accumulation through mechanisms such as oxidative stress, enhanced inflammatory response, and impaired renal excretion.^[22,23]^ Oxidative stress products exacerbate endothelial damage, impair vascular function, and increase microvascular perfusion insufficiency, further suppressing uric acid metabolism and elevating serum uric acid.^[24,25]^ EASIX is also associated with renal dysfunction, and changes in renal function may impair uric acid excretion, exacerbating serum uric acid accumulation.^[22,26]^ Endothelial injury and renal microcirculatory abnormalities directly affect uric acid excretion and enhance uric acid concentration in the bloodstream by altering vascular permeability and renal tubule reabsorption.^[27,28]^ The elevation of EASIX reflects abnormalities in endothelial-platelet interactions, renal function, and cellular metabolism, providing a multidimensional biological framework for understanding the impact of metabolic load on elevated serum uric acid. This mechanism provides a basis for explaining the complex relationship between EASIX and elevated serum uric acid, supporting its potential as a metabolic assessment tool in clinical applications. However, current mechanistic research relies primarily on indirect evidence, lacking direct experimental validation. Future studies should use mechanistic experiments or longitudinal research to explore the specific pathways of each EASIX component in elevated serum uric acid and its potential intervention targets.

This study has several key strengths. First, it used a nationally representative NHANES sample, providing broad generalizability to the US adult population. Second, EASIX is derived from routine laboratory indicators, supporting its feasibility for epidemiological and clinical research. In addition, the use of multivariable adjustment, nonlinear modeling, subgroup analyses, and sensitivity analyses enhanced the robustness of the findings. However, several limitations should be acknowledged. First, because of the cross-sectional design, causal relationships and temporal sequence between EASIX and serum uric acid could not be established, and residual confounding or reverse causality cannot be excluded. Second, EASIX is an indirect marker of endothelial activation and stress and cannot replace direct assessments of endothelial function, such as endothelium-dependent vasodilation. Third, serum uric acid levels may be influenced by recent dietary intake, including purine-rich foods, fructose-containing beverages, alcohol consumption, hydration status, and other short-term metabolic conditions. Because NHANES is an observational survey rather than a controlled feeding study, pre-examination dietary intake could not be fully standardized, and residual dietary confounding may remain. Fourth, this study did not include an independent local or external validation cohort. Although NHANES provides standardized and nationally representative data, validation in prospective cohorts and independent clinical populations is still needed before the findings can be translated into clinical practice. Fifth, EASIX and its individual components are not specific biomarkers of endothelial dysfunction or inflammation. Because platelet count is included in the denominator of EASIX, its inverse pattern across EASIX categories is partly determined mathematically. LDH is a nonspecific marker of cellular and tissue injury, and its tissue source could not be determined using the available data. Direct measurements of endothelial activation, platelet activation or consumption, and systemic inflammation were not available. Therefore, the mechanisms underlying the observed association remain uncertain.

## 5. Conclusion

Among US adults, EASIX was positively and nonlinearly associated with serum uric acid levels. Exploratory analysis further suggested that higher EASIX quartiles were associated with increased odds of hyperuricemia. These findings indicate that EASIX may reflect systemic endothelial stress and metabolic burden related to uric acid variation. Further prospective and externally validated studies are needed to determine its clinical utility.

## Acknowledgments

The authors thank the NCHS and CDC for providing public access to the NHANES database, which was instrumental in completing this study.

## Author contributions

**Conceptualization:** Yujuan Zhu, Yonghui Li.

**Data curation:** Hong Tang, Meng Liu.

**Formal analysis:** Huanyu Wu, Hong Tang, Meng Liu.

**Project administration:** Yonghui Li.

**Resources:** Yujuan Zhu, Yonghui Li.

**Supervision:** Yujuan Zhu, Yonghui Li.

**Writing – original draft:** Huanyu Wu, Hong Tang, Meng Liu.

**Writing – review & editing:** Huanyu Wu, Hong Tang, Meng Liu, Yujuan Zhu, Yonghui Li.




