# Supplementary material for: Association between Endothelial Activation and Stress Index (EASIX) and uric acid levels: A cross-sectional analysis based on the NHANES
Source: Medicine (Baltimore). 2026 Sep 4;105(36):e50443. doi: 10.1097/MD.0000000000050443 (PMC13549515; doi:10.1097/MD.0000000000050443)
Supplement: Supplementary file 1 [file medi-105-e50443-s001.docx]

**Supplementary Table 1 The sensitivity analysis between EASIX and Uric acid.**

|  |  | Model 1  β (95%CI) P-value | Model 2  β(95%CI) P-value | Model 3  β(95%CI) P-value |
| --- | --- | --- | --- | --- |
| Uric acid | EASIX | 1.60 (1.30, 1.80) 0.001 | 0.89 (0.61, 1.20) 0.001 | 0.86 (0.49, 1.20) 0.003 |
|  | Q1 | [Reference] | [Reference] | [Reference] |
|  | Q2 | 0.51 (0.34, 0.67) <0.001 | 0.21 (0.07, 0.35) 0.006 | 0.24 (-0.09, 0.57) 0.087 |
|  | Q3 | 0.94 (0.78, 1.1) <0.001 | 0.44 (0.29, 0.59) <0.001 | 0.46 (0.18, 0.74) 0.019 |
|  | Q4 | 1.30 (1.10, 1.50) <0.001 | 0.70 (0.48, 0.92) <0.001 | 0.72 (0.23, 1.20) 0.024 |
|  | P for trend | <0.001 | <0.001 | <0.001 |

EASIX: endothelial activation and stress index; CI: confidence interval; Q: quartiles;

Model 1: no covariates adjusted; Model 2: adjusted for age, sex, and race; Model 3: adjusted for age, sex, race, BMI, PIR, educational level, smoke, activity status, diabetes, hypertension, CKD.
