# Supplementary material for: Association between Endothelial Activation and Stress Index (EASIX) and uric acid levels: A cross-sectional analysis based on the NHANES
Source: Medicine (Baltimore). 2026 Sep 4;105(36):e50443. doi: 10.1097/MD.0000000000050443 (PMC13549515; doi:10.1097/MD.0000000000050443)
Supplement: Supplementary file 2 [file medi-105-e50443-s002.docx]

**Supplementary Table 2 Sensitivity analysis of the association between EASIX and hyperuricemia.**

|  |  | Model 1  OR (95%CI) P-value | Model 2  OR (95%CI) P-value | Model 3  OR (95%CI) P-value |
| --- | --- | --- | --- | --- |
| **Hyperuricemia** | EASIX | 1.51 (1.13, 2.01) 0.007 | 1.33 (1.03, 1.71) 0.030 | 1.28 (0.93, 1.78) 0.110 |
|  | Q1 | [Reference] | [Reference] | [Reference] |
|  | Q2 | 1.23 (1.00, 1.52) 0.056 | 1.21 (0.96, 1.53) 0.100 | 1.24 (0.67, 2.27) 0.300 |
|  | Q3 | 1.94 (1.45, 2.59) <0.001 | 1.88 (1.34, 2.64) 0.001 | 1.95 (0.94, 4.05) 0.059 |
|  | Q4 | 2.84 (2.11, 3.83) <0.001 | 2.68 (1.89, 3.81) <0.001 | 2.70 (1.13, 6.41) 0.039 |
|  | P for trend | <0.001 | <0.001 | <0.001 |

EASIX: endothelial activation and stress index; CI: confidence interval; Q: quartiles;

Model 1: no covariates adjusted; Model 2: adjusted for age, sex, and race; Model 3: adjusted for age, sex, race, BMI, PIR, educational level, smoke, activity status, diabetes, hypertension, CKD.
